# Morphologic-Molecular Transformation of Oncogene Addicted Non-Small Cell Lung Cancer

**DOI:** 10.3390/ijms23084164

**Published:** 2022-04-09

**Authors:** Fiorella Calabrese, Federica Pezzuto, Francesca Lunardi, Francesco Fortarezza, Sofia-Eleni Tzorakoleftheraki, Maria Vittoria Resi, Mariaenrica Tiné, Giulia Pasello, Paul Hofman

**Affiliations:** 1 Department of Cardiac, Thoracic, Vascular Sciences, and Public Health, University of Padova, 35128 Padova, Italy; federica.pezzuto@unipd.it (F.P.); francesca.lunardi@unipd.it (F.L.); francesco.fortarezza@unipd.it (F.F.); mariaenrica.tine@unipd.it (M.T.); 2 Department of Pathology, Aristotle University of Thessaloniki, 541 24 Thessaloniki, Greece; sofialenatzo@yahoo.com; 3 Department of Surgery, Oncology and Gastroenterology, University of Padova, 35128 Padova, Italy; mariavittoria.resi@iov.veneto.it (M.V.R.); giulia.pasello@unipd.it (G.P.); 4 Medical Oncology 2, Istituto Oncologico Veneto IOV-IRCSS, Padova, 35128 Padova, Italy; 5 Laboratoire de Pathologie Clinique et Expérimentale, FHU OncoAge, Biobank BB-0033-00025, Université Côte d’Azur, 06000 Nice, France; hofman.p@chu-nice.fr

**Keywords:** non-small cell lung cancer, small cell lung cancer, oncogene addicted

## Abstract

Patients with non-small cell lung cancer, especially adenocarcinomas, harbour at least one oncogenic driver mutation that can potentially be a target for therapy. Treatments of these oncogene-addicted tumours, such as the use of tyrosine kinase inhibitors (TKIs) of mutated epidermal growth factor receptor, have dramatically improved the outcome of patients. However, some patients may acquire resistance to treatment early on after starting a targeted therapy. Transformations to other histotypes—small cell lung carcinoma, large cell neuroendocrine carcinoma, squamous cell carcinoma, and sarcomatoid carcinoma—have been increasingly recognised as important mechanisms of resistance and are increasingly becoming a topic of interest for all specialists involved in the diagnosis, management, and care of these patients. This article, after examining the most used TKI agents and their main biological activities, discusses histological and molecular transformations with an up-to-date review of all previous cases published in the field. Liquid biopsy and future research directions are also briefly discussed to offer the reader a complete and up-to-date overview of the topic.

## 1. Introduction

The evolution from pure histological to integrated histological-molecular models in oncology led to the modern concept of precision oncology in which the development of targeted therapies and optimal anticancer treatment is defined on the basis of molecular characterisation of the cancer.

Currently, to treat advanced-stage patients with reimbursed drugs in Europe, four molecular alterations are considered as the minimum requirement in the diagnostic flow of non-small cell lung cancer (NSCLC): epidermal growth factor receptor (*EGFR*) mutations, anaplastic lymphoma kinase (*ALK*), ROS proto-oncogene 1 (*ROS1*) rearrangements, and v-raf murine sarcoma viral oncogene homolog B1 (*BRAF*) mutations [1]. Through different drug access strategies—such as clinical trials, expanded access programs, patient name use programs, and non-negotiated conditions—additional driver alterations have been identified as targets for new therapeutic options: mesenchymal to epithelial transition (*MET*) exon-14 skipping mutations, rearranged during transfection (*RET*) proto oncogene fusions, neurotrophic tyrosine receptor kinase (*NTRK*) fusions, and Kirsten rat sarcoma virus (*KRAS*) G12C mutations [2].

However, despite being effective, several targeted treatments lose efficacy in many oncogene-activated NSCLCs. The mechanisms of these drug resistances are still scarcely known. The aim of this review article is to provide an extensive overview of this topic mainly focusing on morphologic-molecular transformations and their therapeutic implications.

### 1.1. Oncogene Drivers in NSCLC

#### 1.1.1. *EGFR*

The first druggable targets in NSCLC are the sensitising mutations of *EGFR*, occurring in about 12% of NSCLC [1]. Among them, exon-19 deletions and exon-21 point mutations are the two most common gene alterations and are usually targeted by first-(gefitinib, erlotinib), second- (afatinib, dacomitinib), or third- (osimertinib) generation tyrosine kinase inhibitors (TKIs) [3]. Front-line *EGFR* targeting showed a clinical relevant benefit in terms of response rate, of progression-free and of overall survival compared with standard platinum-based chemotherapy [4,5]. The third-generation EGFR TKI osimertinib is currently considered to be the gold standard therapy in naïve *EGFR*-mutant NSCLC, in light of its further prolongation of overall survival, better toxicity profile, and higher intracranial activity compared with other EGFR TKIs [6]. *EGFR* exon-20 insertions are uncommon alterations, which usually do not benefit from first- and second-generation EGFR TKIs, whereas emerging data on osimertinib [7] and new drugs such as poziotinib [8], mobocertinib [9,10], and amivantamab [11] have shown some activity in this subgroup of patients.

Despite the improved efficacy of new-generation TKIs, disease progression still develops through the emergence of mechanisms of acquired resistance, which can be divided into *EGFR*-dependent and *EGFR*-independent mechanisms.

In contrast to the acquired resistance to first- and second-generation TKIs, which is mostly mediated by “on-target” mutations (such as T790M in *EGFR* exon 20), resistance to the third-generation TKI osimertinib has been acquired through more heterogeneous mechanisms, many of them outside the *EGFR* gene [12,13].

The most common *EGFR* tertiary point mutation conferring resistance to osimertinib is C797S in exon-20 [14]. In early studies, fourth-generation EGFR TKIs—such as EAI045 and JBJ-04-125-02—have been found to be active against C797S-T790M-L858R triple mutant NSCLC when given in association with cetuximab or osimertinib, respectively [15,16]. Furthermore, early data suggest the antitumour activity of other fourth-generation EGFR TKIs—such as BBT-176, BLU-945, and TQB3804—in patients with Ex19del/T790M/C797S or L858R/T790M/C797S triple mutant advanced NSCLC [17].

Other *EGFR* tertiary point mutations potentially implicated in resistance mechanisms to osimertinib are C797X, L718Q, L844V, L718V, G796S, G796D, L792, L718, G719, G724, and S768I [13]. The combination of osimertinib with necitumumab seems to be effective [18].

Conversely, loss of the T790M mutation (49%) is usually associated with the activation of different signalling pathways such as *MET* alterations, *KRAS* (G12S, G12D)/ NRAS proto-oncogene (*NRAS)* (E63K) mutations, human epidermal growth factor receptor 2 (*HER2)* amplification, *BRAF* V600E mutation, phosphatidylinositol 3-kinase (*PI3K)* amplification/mutation or phosphatase and tensin homolog (*PTEN)* deletion, cell-cycle gene alterations, and oncogenic fusions [13]. Among them, *MET* amplification is the most common (7–15%; 5–50%) [19] and combining MET inhibitors (e.g., crizotinib) with osimertinib has been found to be effective in this setting [20]. Other studies are testing the efficacy and safety of combining osimertinib with anti-MET (savolitinib) or anti- mitogen-activated protein kinase kinase 1 and 2 (*MEK1/2*) (selumetinib) TKIs in *EGFR*-mutant patients progressing to previous EGFR TKIs [21].

Several ongoing phase II/III trials (ORCHARD, INSIGHT, SAVANNAH, and GEOMETRY) [18,22,23,24,25,26,27] are investigating combinations of targeted therapies in *EGFR*-mutant advanced NSCLC patients whose disease has progressed after osimertinib administration.

Other off-target resistance mechanisms are gene fusions (1–8%; 3–10%), particularly *ALK-* or *RET-*fusions, and *RAS-* Mitogen-Activated Protein Kinase *(MAPK)* pathway aberrations (3–4%; 2–8%) [13]. The phase II ORCHARD trial is evaluating the efficacy and safety of combining osimertinib with ALK inhibitors (alectinib), RET inhibitors (selpercatinib) or MEK1/2 inhibitors (selumetinib) in these subgroups of patients [18].

Interestingly, overexpression of human epidermal growth factor receptor (*HER) 2* and *HER3* is detectable in several *EGFR*-mutant NSCLCs and is associated with worse clinical outcomes. A phase I trial is evaluating the antitumour activity and safety profile of patritumab deruxtecan, an HER3 antibody, in *EGFR*-mutant NSCLC patients progressing to a prior EGFR TKI and platinum-based chemotherapy. This new drug seems to be active against different mechanisms of resistance, including *EGFR* C797S mutation, *BRAF* fusion, and *MET* and *HER2* amplification [28].

In those cases where *HER2* amplification/overexpression occurs (1–2%; 5%), combining osimertinib with trastuzumab deruxtecan was found to be effective [TRAEMOS] [13,29].

Lastly, early studies suggest that using triple *EGFR/BRAF/MEK* pathway co-inhibition (osimertinib + dabrafenib + trametinib) has potential for overcoming acquired resistance mediated by the activation of *BRAF* pathways [30].

#### 1.1.2. *ALK*

*ALK-* echinoderm microtubule-associated protein-like 4 *(EML4)* rearrangements in chromosome 2 occur in about 5% of cases [31]. First-(crizotinib) and second-(ceritinib, alectinib, brigatinib) generation TKIs are approved for the front-line treatment of ALK-positive advanced NSCLC patients, in light of their positive effect on survival compared to chemotherapy [32,33]. Second-generation alectinib and brigatinib have currently become the standard of care over crizotinib, due to their superior safety profile and their better intracranial activity [34,35,36]. The third-generation ALK TKI lorlatinib is currently the indicated second-line therapy for ALK-positive NSCLC progressing to alectinib, ceritinib or crizotinib [37].

In contrast to the *EGFR* story, the treatment sequence in ALK positive lung cancer has been defined independently by the detection of a specific resistance mechanism. However, the probability of selected *ALK* mutations as acquired resistance drivers increases after the last-generation ALK TKIs [38], making them a potential criteria in the selection of subsequent lines of treatment. Secondary *ALK* mutations occur in approximately 50% of cases of resistance to second-generation ALK TKIs. Among them, the most common is G1202R (35–60%) with lorlatinib being the only ALK TKI that which seems to be active against this point mutation [39]. Other drugs (anti ALK I1171N and ALK F1174V) have been used to mediate resistance to the first, second and third generations of ALK TKI [38,40].

Off-target mechanisms of resistance include bypass signalling (such as *EGFR, MET*, *c-KIT, SRC*, *RAS/MAPK,* and Src-homology 2 domain-containing phosphatase 2-*SHP2* mutations). *MET* amplification has been identified in 15% of patients progressing to ALK inhibitors [41]. According to preclinical evidence, patients with acquired *MET* alterations may benefit from combining the ALK selective inhibitor lorlatinib with a MET inhibitor (e.g., capmatinib—particularly in patients with central nervous system disease, savolitinib, and crizotinib) [42].

#### 1.1.3. *ROS1*

*ROS1* rearrangements occur in 1–2% of advanced NSCLC. Given the positive results from several studies [43], crizotinib has been approved by the food and drug administration (FDA) and european medicines agency (EMA) as a front-line therapy in *ROS1*-rearranged advanced NSCLC. However, drug resistance and progressive disease—including central nervous system progression—inevitably occur [44]. Novel TKIs (entrectinib, lorlatinib, repotrectinib) and some other ongoing studies have shown good intracranial activity in patients with treatment-naïve *ROS1*-rearranged NSCLC [45,46].

Regarding *ROS1*-dependent mechanisms of acquired resistance, *ROS1* secondary point mutations (G2032R, D2033N, L2026M, S1986F/Y) occur in approximately 50−60% of crizotinib resistant NSCLC cases [47]. Among next-generation ROS1 TKIs, lorlatinib seems to be active against K1991E and S1986F mutations but has shown no efficacy against G2032R and L2026M mutations [48], whereas repotrectinib has shown activity against G2032R [46].

*ROS1*-independent resistance mechanisms may include the activation of other signalling pathways such as *EGFR*, *MET*, *HER2*, *KRAS*, *KIT*, *BRAF*, and *MEK* [44]. Preclinical studies suggest combination therapies as a strategy to overcome this category of acquired resistance to crizotinib, although clinical results are not yet available [49].

#### 1.1.4. *RET*

*RET* fusions are found in 1–2% of NSCLC [1] and the most common patterns of fusions are kinesin family member 5B (*KIF5B)-RET* (70–90%) and coiled-coil domain containing 6 (*CCDC6)-RET* (10–25%) [50]. Multitarget TKIs (cabozantinib, lenvatinib, vandetanib, and ponatinib) have shown modest activity and high toxicity in advanced *RET* fusion-positive NSCLC patients [51,52,53,54]. Recently, some trials have shown clinical efficacy and a good safety profile of new highly selective RET inhibitors, pralsetinib and selpercatinib [55,56]. Based on these good outcomes, they have been approved by the FDA as a front-line therapy for the treatment of advanced *RET* fusion-positive NSCLC patients [55,56]. In Europe, these drugs are approved by the EMA as a second-line treatment after a first-line treatment based on immunotherapy or an immune-chemotherapy [55].

Despite the encouraging efficacy of these new drugs, acquired mechanisms of resistance will probably limit the duration of their clinical benefit. The majority of cases progressing to RET selective inhibitors are driven by *RET*-independent mechanisms, particularly *MET* amplification (15%). Further studies will be required to assess the safety and efficacy of combining RET and MET inhibitors or of administering a multitarget TKI with both anti-MET and anti-RET activity (e.g., cabozantinib) [57].

Regarding on-target mechanisms, more data are needed to elucidate whether the different *RET*-secondary point mutations (e.g., G810C, G810S, V804) are more likely to confer resistance to selpercatinib and/or pralsetinib [57].

#### 1.1.5. *MET*

*MET* amplification and *MET* exon-14 skipping mutation occur in 1–3% and 3–4% of NSCLC, respectively [1]. Currently, MET TKIs (capmatinib, tepotinib, crizotinib) represent the standard front-line therapy in *MET*-positive advanced NSCLC according to the National Comprehensive Cancer Network Guidelines [58]. Despite the improved efficacy of MET TKIs, progression of disease occurs in 75% of patients under treatment [59].

On-target mechanisms of resistance (35%) include *MET* amplification and *MET* kinase domain mutations. D1228, Y1230, H1094, G1163, and L1195 point mutations frequently mediate resistance to type I (crizotinib, capmatinib) and type II (glesatinib) MET TKIs, whereas D1228 and Y1230 point mutations are likely to confer resistance to type I MET TKIs [59].

Off-target mechanisms of resistance (45%) include *EGFR* amplification (frequently involved in resistance to type I MET TKIs), *KRAS* mutations, and *BRAF* amplifications [59]. New therapeutic combination strategies are required to bypass secondary resistance in this subgroup of patients.

#### 1.1.6. *BRAF*

Up to 5% of lung adenocarcinomas develop *BRAF*-activating mutations [60]. Considering the positive results observed in clinical trials, dabrafenib combined with trametinib has currently become the standard of care in advanced *BRAF*-V600E NSCLC [61]. However, disease progression to BRAF/MEK inhibitors still occurs due to the development of acquired resistance mechanisms, frequently associated with the persistence of the *BRAF*-V600E driver mutation. Potential mechanisms of resistance are: *KRAS* (Q61R, G12V)/*NRAS* (Q61R, Q61K) mutations; activation of *PI3K-AKT-*mechanistic target of rapamycin *(mTOR)* and *RAS-RAF-MEK* pathways (*MEK1 K57N*, *PTEN N329fs*), and isocitrate dehydrogenase (NADP (+)) 1(*IDH1);* U2 small nuclear RNA auxiliary factor 1 *(U2AF1);* and catenin beta 1 (*CTNNB1)* alterations [62,63,64,65].

Novel treatments and combination strategies to overcome acquired resistance are needed.

#### 1.1.7. *KRAS*

*KRAS* mutations are the most common oncogenic drivers in NSCLC (20–25%), and among them, *KRAS*-G12C is the most frequent (50%) [66]. Despite phase I-III studies demonstrating the efficacy of KRAS-G12C inhibitors, such as sotorasib (CodeBreak 100, 200) and adagrasib (KRYSTAL-1, -12), up to 50–60% of patients do not respond to them [67]. Other phase I studies are evaluating the activity of different KRAS TKIs (GDC-6036 [NCT04449874], JNJ-74699 [NCT04006301] and D-1553 [NCT04585035]).

Resistance to KRAS-G12C TKIs can be mediated by secondary *KRAS* mutations (e.g., Y40A, N116H, or A146V; A59G, Q61L, or Y64A) [68] or activation of different signalling pathways by other protein alterations (e.g., *SHP2* [69], SOS Ras/Rac guanine nucleotide exchange factor 1 (*SOS1)* [70], aurora kinase A (*AURKA)* [71], or *EGFR/fibroblast growth factor receptor (FGFR)/PI3K* [72]).

Several ongoing studies are considering the efficacy and tolerability of combination therapies to overcome these mechanisms of resistance, associating KRAS-G12C inhibitors with other small molecules—such as SHP2 inhibitors (TNO155, RMC-4630), SOS1 inhibitor (BI 1701963), EGFR inhibitors (panitumumab, cetuximab, afatinib), CDK4/6 inhibitors (palbociclib), MEK inhibitor (trametinib ± panitumumab) or mTOR inhibitor (everolimus). However, clinical data about therapeutic strategies to overcome acquired resistance in this setting of patients are still unavailable.

#### 1.1.8. *NTRK*

*NTRK* fusions are found in less than 1% of advanced NSCLC [73]. First-generation TRK inhibitors, larotrectinib and entrectinib, are currently indicated as first-line therapy in NTRK-positive NSCLC [74,75]. The emergence of mechanisms of resistance to TRK inhibitors is a direct consequence of the development of secondary mutations (e.g., NTRK1-G667C, NTRK3-G696A, and xDFG mutations) or other genomic *MAPK* pathway alterations (including *BRAF*-V600E, *KRAS*-G12D, and *MET* amplification) [73]. Early clinical trials have evaluated the antitumour activity of new-generation TRK-inhibitors, such as selitrectinib [76] and repotrectinib [77], in NTRK-positive NSCLC patients who develop acquired resistance mutations to larotrectinib or entrectinib. Further studies are required to investigate possible therapeutic strategies to overcome the resistance mechanisms of TRK inhibitors in TRK-positive NSCLC.

**Figure 1:**
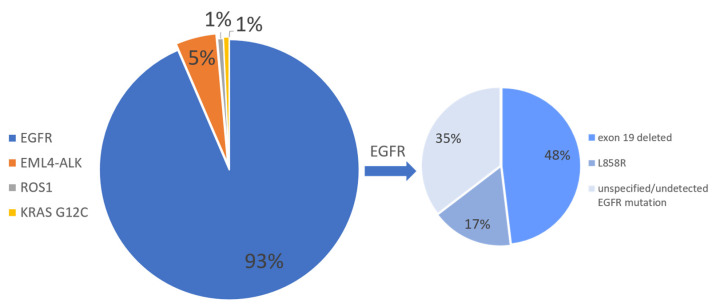
The pie chart shows the percentage of cases transformed for each mutational status.

Phenotypical changes have been reported as an important mechanism of resistance in many NSCLC harbouring all the above-mentioned driver mutations (Figure 1).

## 2. Morphological and Molecular Transformation

### 2.1. High-Grade Neuroendocrine Carcinomas

**Figure 2:**
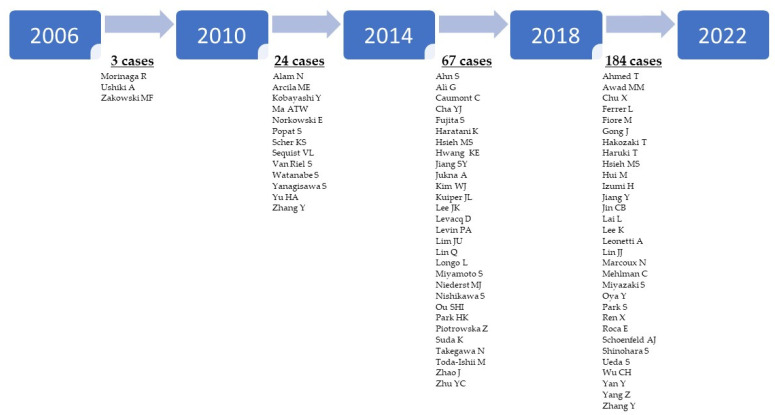
Timeline of switched oncogene-addicted NSCLCs. The timeline shows a significant increase in the number of switched oncogene-addicted NSCLC reported in the literature from 2006 to 2022.

Histologic transformation in high-grade neuroendocrine carcinomas, mainly small cell lung carcinoma (SCLC), has been reported to be the most common form. The first case was reported by Zakowski et al. in 2006 [78]. The authors described a histological transformation of *EGFR*-mutant lung adenocarcinoma in a middle-aged never smoker woman treated with TKI. Histologic examination of the biopsy at recurrence and of multiple metastases at autopsy showed unambiguous morphological and immunohistochemical features of SCLC without any foci of adenocarcinoma. Even more interesting was that the original exon 19 deletion of the *EGFR* gene was retained. Before 2010, only one similar case was reported [79]. The body of evidence in this direction has increased in the last decade (Figure 2).

Most SCLCs supposed to be switched from a NSCLC have occurred in *EGFR*-mutated tumours [80,81,82,83,84,85,86,87,88,89,90,91,92,93,94,95,96,97,98,99,100,101,102,103,104,105,106,107,108,109,110,111,112,113,114,115,116] (Table 1, Table 2 and Table 3). Many publications were single case reports or rare case series, whereas only a few studies were carried out on wider populations (Table 1, Table 2 and Table 3).

**Figure 3:**
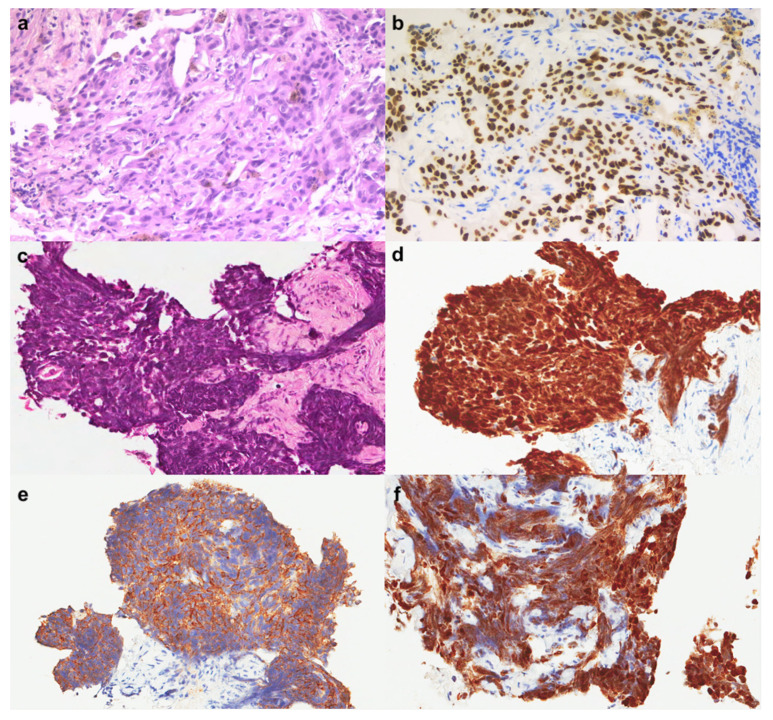
Index case of switched lung adenocarcinoma from Padova case series. The patient was a 40-year-old male, ex-light smoker (6 packs/year) with a right hilar pulmonary mass with bone metastasis. A transbronchial biopsy was performed and a lung adenocarcinoma with TTF1 expression was diagnosed: (**a**) haematoxylin and eosin, original magnification ×200; (**b**) TTF1 immunostaining, original magnification ×200. Molecular analyses showed exon 19 deletion of EGFR for which II line TKI therapy was implemented. One year later the patient presented disease progression with lung, bone, and brain metastases. A new transbronchial biopsy was performed showing a poorly differentiated neoplasm whose morphological and immunohistochemical characteristics were consistent with a SCLC: (**c**) haematoxylin and eosin, original magnification ×100; (**d**) TTF1, immunohistochemistry, original magnification ×200; (**e**), synaptophysin, immunohistochemistry, original magnification ×200; (**f**) Ki67, immunohistochemistry, original magnification ×200. These findings were consistent with a small cell lung cancer.

Morphological features and immunohistochemical phenotypes of switched SCLC are those typically detected in such histotypes, with positivity of neuroendocrine markers, mainly synaptophysin and chromogranin. This is also the case in our research center where six histological switches were diagnosed from 2017 to 2021 [117] (Figure 3).

Aggressive clinical behaviour and poor prognosis are also superimposable with naïve SCLC, while epidemiological distribution shows a high incidence in non-smoker or former-smoker patients [118].

Although systematic studies concerning the latency between the start of treatment and the phenotype switch in EGFR-mutant adenocarcinoma are lacking, in the large series of cases studied by Ferrer [119] and Marcoux [111] the median time to transformation was 16 months and 17.8 months, respectively.

Although the histologic transformation is now recognised, there are still many aspects and issues that need to be resolved. The most debated issues concern the molecular mechanisms responsible for the switch and the consequent possible therapeutic strategies that could be adopted to treat or even prevent switched SCLC. One of the most frequent hypotheses identifies the coexistence of adenocarcinoma and SCLC already at the onset of the neoplasm. In this scenario, SCLC gradually takes over the other histotype under the pressure of TKI. This hypothesis has its roots in two studies in the late eighties by Adelstein [120] and Mangum [121] in which the authors described NSCLC foci in the re-biopsy of patients affected by SCLC. It is a plausible hypothesis, especially considering that the first diagnosis of the neoplasm occurred on small biopsies or cytologic samples, for which the sampling error and the inadequacy of material may have constituted the main problem of tumour representation. However, combined histology would not explain why, in its different combined forms, the response of patients to TKI is initially good regardless of the presence of SCLC and subsequently becomes dramatic when SCLC is diagnosed [122]. The most recent molecular acquisitions have highlighted other mechanisms that are probably more suited to interpret such a complex process.

Another hypothesis is that a transformation from NSCLC to SCLC may actually occur. A point in favour of this possibility is that almost all SCLCs that arose from *EGFR*-mutant adenocarcinoma retained the molecular signature, i.e., an *EGFR* activating mutation [123]. The transformation hypothesis is also supported by molecular alterations affecting genes strictly typical of SCLC. Although molecular mechanisms that determine the onset of a neuroendocrine tumour are complex and heterogeneous [124], a high prevalence of *TP53* and *retinoblastoma protein (Rb)1* mutations has been identified in SCLC genome-sequencing studies [125,126,127], suggesting their pivotal role in the development and progression of the disease. This is almost certain given that, even in switched SCLC, Rb1 was lost in 100% of cases [88] and was the substrate of the trial (NCT03567642) for the upfront use of TKI and platinum/etoposide-based chemotherapy in adenocarcinoma carrying the triple mutation *EGFR-Rb-p53*. Similarly, active proliferation, which is essentially the target of cytotoxic chemotherapies, is also the target of B-cell lymphoma 2 (Bcl-2) inhibitors, whose administration has recently been studied in switched SCLCs [88]. Moreover, molecular alterations in cell cycle processes strictly related to Rb1 loss may also be vulnerable to the action of new therapeutic targets [128,129,130].

Although these mutations are necessary, they are insufficient for the acquisition of resistance and for neuroendocrine differentiation, as also indicated by experimental studies performed in *Rb1* knockdown *EGFR*-mutant cell-lines [131]. This consideration raises another question, namely: what other factors can contribute to histotype transformation? It may be that the inactivation of *Rb1* is associated with other mechanisms in an in vivo context. For instance, Meder et al. [131] showed that *Rb1* inactivation could derive from a phosphorylation by cyclin-dependent kinase 5 (CDK5), which is in turn activated by the achaete-scute family BHLH transcription factor 1 (ASCL1) overexpression, a transcription factor involved in neuroendocrine differentiation and regulated by the NOTCH pathway.

Other transcriptional and epigenetic events may influence genetic modifications, making neuroendocrine cells even more like classic SCLC [88]. The different expression of miRNAs is an example in this direction. Indeed, switched SCLC miRNA analyses have demonstrated the upregulation of miRNA subtypes typical of de novo SCLC and the simultaneous expression of the miRNA subtypes detected in adenocarcinoma, making this form of SCLC a somewhat hybrid tumour [127].

This field is as unexplored as it is fascinating, even from a therapeutic perspective, giving the chance to modulate epigenetic events with curative intentions towards switched SCLC and preventive measures before histologic transformation [132].

The assumption that NSCLC may switch histology into SCLC raises the possibility of a common cell of origin with the ability to differentiate into both histotypes. Starting from the results of a few murine models, the focus was mainly on alveolar type II cells that initially were supposed to potentially develop both SCLC and *EGFR*-mutant adenocarcinoma [122]. From the first evidence of transcriptome analyses, alveolar type II cells showed high expression of *EGFR* family members, thus assigning a key role to *EGFR* towards this lineage differentiation.

However, a switch in SCLC also occurs in lung adenocarcinoma with other driver mutations such as *ALK* [133,134,135,136,137,138,139,140,141] and *ROS1* [142,143] foreshadowing possible more complex mechanisms (Table 4 and Table 5). In addition, more efforts should be made to study rare events, such as the transformation in LCNEC, occasionally described in case reports [109,144,145,146]. Although SCLC and LCNEC share some peculiarities, such as the molecular substrate, they represent two distinct entities. Thus, comprehensive studies are warranted.

**Table 1:** Histological transformation in EGFR exon 19 deleted NSCLC (adenocarcinoma).

First Author	Year/Journal	Number of Cases	Sex	Age (Mean Year)	Smoking Habits (Yes/No)/Number of Smokers	TKI Treatment Type	Other Treatment (Yes/No) *	Histological Transformation (Lung or Metastatic Sites)
Morinaga, R. [79]	2007/Lung Cancer	1	F	46	No	Ist	Yes	SCLC
Ushiki, A. [147]	2009/Jpn. J. Clin. Oncol.	1	M	58	No	Ist	Yes	PLC
Sequist, V.L. [81]	2011/Sci. Transl. Med.	2 of 20	F	47	NA	Ist	No	SCLC
Van Riel, S. [82]	2012/Ann. Oncol.	1	F	42	No	Ist	Yes	SCLC
Yanagisawa, S. [146]	2012/Respirology	1	M	46	No	Ist	Yes	LCNEC
Norkowski, E. [83]	2013/J Thorac. Oncol.	2 of 5	F	55	No	Ist	Yes/No	SCLC/Mixed AC + SCLC
Popat, S. [84]	2013/Lung Cancer	1	F	46	No	Ist	Yes	Combined NSCLC + SCLC
Scher, K.S. [148]	2013/J. Natl. Compr. Canc. Netw.	1	F	58	Yes/1	Ist	Yes	SCC
Watanabe, S. [85]	2013/Lung Cancer	1	F	52	No	Ist	Yes	SCLC
Zhang, Y. [86]	2013/Lung Cancer	1	M	80	No	Ist	No	SCLC
Lim, J.U. [145]	2014/Korean J. Intern. Med.	1	M	33	ΝA	Ist	Yes	AC + LCNEC
Hsieh, M.S. [149]	2015/Ann. Thorac. Surg.	1	F	51	NS	Ist	No	SCC
Hwang, K.E. [87]	2015/Tumori. J.	1	M	61	Yes/1	Ist	No	SCLC
Levin, P.A. [150]	2015/J. Thorac. Oncol.	1	F	66	No	Ist	Yes	SCC
Niederst, M.J. [88]	2015/Nat. Commun.	6	4F/2M	56.5	NA	Ist	No	SCLC
Piotrowska, Z. [106]	2015/Cancer Discov.	1	F	46	NA	Ist/IIIrd **	No	SCLC
Suda, K. [89]	2015/Sci. Reports	1 of 16	F	76	No	Ist *	Yes	AC + SCLC
Ahn, S. [151]	2016/J. Pathol. Transl. Med.	3	F	56	No	2 Ist/1 IIst	2 Yes/1 No	1 SCLC/2 combined AC + SCLC
Alì, G. [91]	2016/Oncol. Lett.	1	M	45	No	Ist	Yes	SCLC
Haratani, K. [152]	2016/Ann. Oncol.	1	F	48	No	Ist	Yes	SCC
Jukna, A. [153]	2016/J. Thorac. Oncol.	1	F	79	No	Ist	Yes	SCC
Lee, J.K. [92]	2017/J. Clin. Oncol.	21	15F/6M	55	No	17 Ist/6 IInd/8 IIIrd	7 Yes/15 No	16 SCLC/5 AC + ACLC
Park, H.K. [154]	2017/J. Pathol. Transl. Med.	1	M	40	Yes	IInd	Yes	SCC
Chu, X. [94]	2018/J. Thorac. Oncol.	1	F	58	No	Ist	Yes	SCLC
Ferrer, L. [119]	2018/J. Thorac. Oncol.	36	NS	NS	NS	NS	NS	SCLC
Hui, M. [95]	2018/Lung India	1	M	46	Yes/1	Ist	Yes	SCLC
Zhao, J. [93]	2018/Thorac. Cancer.	5	4F/1M	53.6	Yes/1	Ist	2 Yes/1 No	SCLC
Fiore, M. [96]	2019/Tumori. J.	1	F	56	Yes/1	Ist	Yes	SCLC
Park, S. [155]	2019/Lung Cancer	3	M	54.3	NS	1 Ist/2 IInd	1 Yes/2 No	SCC
Hakozaki, T. [97]	2020/Intern. Med.	1	F	70	No	Ist	Yes	Mixed SCC + SCLC
Haruki, T. [156]	2020/Mol. Clin. Oncol.	1	F	56	No	Ist/IIIrd	Yes	SCC
Hsieh, M.S. [157]	2019/Lung Cancer	3	F	50	No	3 Ist/2 IInd/1 IIIrd	Yes	AC with sarcomatoid transformation
Miyazaki, S. [144]	2020/J. Clin. Med. Case Rep.	1	M	64	Yes/1	IInd/IIIrd	Yes	LCNEC
Ren, X. [98]	2020/J. Int. Med. Res.	1	M	52	No	Ist/IIIrd	Yes	SCLC
Schoenfeld, A.J. [99]	2020/Clin. Cancer Res.	7	NS	NS	NS	IIIrd	No	3 SCLC/3 SCC/1 PLC
Yan, Y. [158]	2020/Clinic Lung Cancer	1	M	58	Yes/1	Ist/IIIrd **	Yes	AC with sarcomatoid features
Jiang, Y. [115]	2021/Medicine	1	M	54	Yes/1	Ist	No	Mixed AC + SCLC
Jin, C.B. [80]	2021/World J. Clin. Cases	6	3F/3M	54.5	Yes/2	6 Ist/2 IIIst	No	5 SCLC/1 LCNEC
Lai, L. [101]	2021/Medicine	2	F/M	41	Yes/1	Ist/IIIrd	Yes	SCLC
Leonetti, A. [116]	2021/Front. Oncol.	1	F	63	No	Ist/IIIrd	No	SCLC
Yang, Z. [102]	2021/J. Int. Med. Res.	1	M	57	Yes/1	Ist	No	SCLC

ASCC: adenosquamous carcinoma; AC: adenocarcinoma; F: female; LCNEC: large cell neuroendocrine carcinoma; M: male; NA: not available; NS: not specified; NSCLC: non-small cell lung carcinoma; PLC: pleomorphic lung carcinoma; SCC: squamous cell carcinoma; SCLC: small cell lung carcinoma; TKI: tyrosine kinase inhibitor. * chemotherapy/radiotherapy/immunotherapy; ** associated with T790M mutation.

**Table 2:** Histological transformation in *EGFR* L858R mutated NSCLC (adenocarcinoma).

First Author	Year/Journal	Number of Cases	Sex	Age (Mean Year)	Smoking Habits (Yes/No)/Number of Smokers	TKI Treatment Type	Other Treatment (Yes/No) *	Histological Transformation (Lung or Metastatic Sites)
Alam, N. [103]	2010/Clin. Lung Cancer	1	F	73	No	Ist	Yes	SCLC
Sequist, V.L. [101]	2011/Sci. Transl. Med.	4	3F/1M	59.5	NA	Ist	No	3 SCLC/1 SC
Ma, A.T.W. [104]	2012/Acta Oncol.	1	F	65	No	Ist	No	SCLC
Hsieh, M.S. [149]	2015/Ann. Thorac. Surg.	1	F	61	No	Ist	Yes	SCC
Jukna, A. [153]	2016/J. Thorac. Oncol.	1	F	74	Yes/1	Ist	No	SCC
Kim, W.J. [105]	2015/Thorac. Cancer	1	M	73	No	Ist	Yes	SCLC
Kuiper, J.L. [159]	2015/J. Clin. Pathol.	1	F	63	No	Ist	Yes	SCC
Niederst, M.J. [88]	2015/Nat. Commun.	2	F	59.5	NA	Ist	No	SCLC
Piotrowska, Z. [106]	2015/Cancer Discov.	1	F	52	NA	Ist/IIIrd	No	SCLC
Toda-Ishii, M. [160]	2015/Int. J. Clin. Exp. Pathol.	1	F	72	No	Ist	Yes	AC with sarcomatous transformation
Ahn, S. [90]	2016/J. Pathol. Transl. Med.	1	F	57	No	Ist/IIst	Yes	SCLC
Jiang, S.Y. [100]	2016/Medicine	1	M	46	No	Ist	Yes	SCLC
Lin, Q. [107]	2016/BMC Cancer	1	M	49	Yes/1	Ist	Yes	SCLC
Nishikawa, S. [108]	2016/Ann. Oncol.	1	F	76	Yes/1	Ist	No	SCLC
Haratani, K. [152]	2016/Ann. Oncol.	1	F	64	No	Ist **	Yes	SCC
Lee, J.K. [92]	2017/J. Clin. Oncol.	4	3F/1M	58.8	Yes/2	4 Ist/1 IIIrd	2 Yes/2 No	2 AC + SCLC/2 SCLC
Longo, L. [161]	2017/Lung Cancer	1	F	43	Yes/1	Ist	Yes	Mixed SCC + AC
Ferrer, L. [119]	2018/J. Thorac. Oncol.	36	NS	NS	NS	NS	NS	SCLC
Izumi, H. [162]	2018/Clinic Lung Cancer	1	M	68	Yes/1	Ist	Yes	SCC
Shinohara, S. [163]	2018/J. Thorac. Disease	1	M	62	No	Ist	No	SCC
Zhao, J. [93]	2018/Thorac. Cancer	2	M	59	No	Ist	Yes/No	SCLC
Park, S. [155]	2019/Lung Cancer	1	F	65	NS	Ist/IIIrd	No	ASCC
Roca, E. [164]	2019/Lung Cancer	1	F	67	NS	Ist/IIIrd	No	SCC
Hsieh, M.S. [157]	2019/Lung Cancer	2	F	65	No	2 Ist/1 IIst/1 IIIrd	Yes	AC with sarcomatoid transformation
Schoenfeld, A.J. [99]	2020/Clin. Cancer Res.	1	NS	NS	NS	IIIrd	No	SCC

ASCC: adenosquamous carcinoma; AC: adenocarcinoma; F: female; M: male; NA: not available; NS: not specified; PLC: pleomorphic lung carcinoma; SC: sarcomatoid carcinoma; SCC: squamous cell carcinoma; SCLC: small cell lung carcinoma; TKI: tyrosine kinase inhibitor. * chemotherapy/radiotherapy/immunotherapy; ** associated with T790M mutation.

**Table 3:** Histological transformation in unspecified *EGFR*-mutated NSCLC and in EGFR TKI-treated NSCLC with undetected mutation.

First Author	Year/Journal	Number of Cases	Sex	Age (Mean Year)	Smoking Habits (Yes/No)/Number of Smokers	TKI Treatment Type	Other Treatment (Yes/No) *	Histological Transformation (Lung or Metastatic Sites)
Zakowski, M.F. [78]	2006/N. Engl. J. Med.	1	F	45	No	Ist	Yes	SCLC
Arcila, M.E. [109]	2011/Clin. Cancer Res.	3	NA	NA	NA	NS	No	2 SCLC/1 LCC and HGNEC
Yu, H.A. [114]	2013/Clin. Cancer Res.	4	NA	NA	NA	Ist	NA	SCLC
Ahn, S. [110]	2016/J. Pathol. Transl. Med.	1	F	68	No	Ist	Yes	SCLC
Ahmed, T. [110]	2018/Lung Cancer	8	NS	NS	NS	NS	No	SCLC
Ferrer, L. [119]	2018/J. Thorac. Oncol.	36	NS	NS	NS	NS	NS	SCLC
Lee, K. [112]	2019/Lung Cancer	6	NS	NS	NS	1 Ist/5 IInd	No	3 SCLC/3 SCC
Marcoux, N. [111]	2019/J. Clin. Oncol.	58	NS	NS	NS	NS	NS	SCLC
Mehlman, C. [113]	2019/Lung Cancer	5	NS	NS	NS	IIIrd	No	4 SCLC/1 SCC
Schoenfeld, A.J. [99]	2020/Clin. Cancer Res.	1	NS	NS	NS	IIIrd	No	SCC
Jin, C.B. [80]	2021/World J. Clin. Cases	3	1F/2M	60.7	Yes/2	3 Ist/1 IIIrd	Yes	2 SCLC/1 SCC

F: female; HGNEC: high grade neureoendocrine carcinoma; LCC: large cell carcinoma; M: male; NA: not available; NS: not specified; SCC: squamous cell carcinoma; SCLC: small cell lung carcinoma; TKI: tyrosine kinase inhibitor. * chemotherapy/radiotherapy/immunotherapy.

**Table 4:** Histological transformation in *EML4-ALK* mutated NSCLC (adenocarcinoma).

First Author	Year/Journal	Number of Cases	Sex	Age (Mean Year)	Smoking Habits (Yes/No)/Number of Smokers	TKI Treatment Type	Other Treatment (Yes/No) *	Histological Transformation (Lung or Metastatic Sites)
Kobayashi, Y. [165]	2013/J. Thorac. Oncol.	1	M	32	NS	Ist	Yes	SC
Cha, Y.J. [133]	2016/J. Thorac. Oncol.	1	M	72	No	Ist	Yes	SCLC
Fujita, S. [135]	2016/J. Thorac. Oncol.	1	F	67	No	Ist	Yes	SCLC
Caumont, C. [134]	2016/Lung Cancer	1	F	63	No	Ist	Yes	NSCLC with neuroendocrine morphology
Levacq, D. [136]	2016/Lung Cancer	1	F	53	No	Ist	Yes	SCLC
Miyamoto, S. [137]	2016/Jpn. J. Clinic. Oncol.	1	F	56	No	Ist/IInd	Yes	SCLC
Takegawa, N. [138]	2016/Ann. Oncol.	1	F	43	NS	Ist/IInd	Yes	SCLC
Ou, S.H.I. [139]	2017/Lung Cancer	1	M	35	No	IInd/IIIrd	No	SCLC
Zhu, Y.C. [140]	2017/Onco. Targets Ther.	1	M	49	No	Ist	Yes	SCLC
Oya, Y. [141]	2018/Oncol. Lett.	1	M	62	Yes/1	IInd	Yes	SCLC
Gong, J. [166]	2019/J. Natl. Compr. Canc. Netw.	1	F	60	Yes/1	Ist/IInd	Yes	SCC
Park, S. [155]	2019/Lung Cancer	1	F	52	No	Ist/IInd	No	SCC
Ueda, S. [167]	2021/Thorac. Cancer	1	F	58	Yes/1	Ist/IInd/IIIrd	Yes	SCC
Zhang, Y. [168]	2021/Pathol. Oncol. Res.	1	F	47	No	Ist/IInd/IIIrd	No	SCC

F: female; M: male; NS: not specified; NSCLC: non-small cell lung carcinoma; SCC: squamous cell carcinoma; SCLC: small cell lung carcinoma; TKI: tyrosine kinase inhibitor. * chemotherapy/radiotherapy/immunotherapy.

**Table 5:** Histological transformation in NSCLC (adenocarcinoma) with other mutations.

First Author	Year/Journal	Number of Cases	Sex	Age (Mean Year)	Smoking Habits (Yes/No)/Number of Smokers	Mutation	TKI Treatment Type	Other Treatment (Yes/No) *	Histological Transformation (Lung or Metastatic Sites)
Lin, J.J. [142]	2020/NPJ. Precis. Oncol.	1	F	32	NA	*ROS1*	Ist/IIIrd	Yes	SCLC
Awad, M.M. [169]	2021/N. Engl. J. Med.	2	ΝA	ΝA	ΝA	*KRAS G12C*	Ist	No	SCC
Wu, C.H. [143]	2021/Thorac. Cancer	1	M	63	NA	*ROS1*	Ist	Yes	SCLC

F: female; M: male; NA: not available; SCC: squamous cell carcinoma; SCLC: small cell lung carcinoma; TKI: tyrosine kinase inhibitors. * chemotherapy/radiotherapy/immunotherapy.

### 2.2. Squamous Cell Carcinoma (SCC)

Few cases of oncogene-addicted adenocarcinomas that switched into SCC have been reported. Certainly, this phenomenon is less common than neuroendocrine transformation [118]. However, the incidence has been progressively increasing in recent years mainly during first-line osimertinib compared with the later-line osimertinib or earlier generation EGFR TKIs [99,154].

Although this transformation has mainly been thought to arise from a clonal selection of pre-existing subclones, the possible occurrence of a true lineage shift in this entity cannot presently be excluded. From a morphological point-of-view, there are no particular histological aspects that characterise the switching of adenocarcinomas into SCC, and in all cases the SCC histotype was confirmed by p40 or p63 positive immunostaining [80,97,99,112,113,148,149,150,152,153,155,156,159,161,162,163,164,166,167,168,169]. Interestingly, the biological/molecular bases of this transformation have not yet been clarified; indeed, no consistent genomic signature has been identified despite comprehensive genomic analyses. However, some clinical and experimental studies have reported important information.

Jin et al. performed the first genomic profiling of *EGFR*-mutant SCC and reported a higher mutation frequency of neurofibromatosis (Neurofibromatosis type 1-*NF1*), ataxia telangiectasia and Rad3 (*ATR*) related gene, and breast cancer (BReast CAncer gene 1-*BRCA1*) compared with *EGFR*-mutant adenocarcinomas [170]. However, the authors did not find any information about the transformation of adenocarcinoma in SCC, because the two histotypes did not occur in the same patients as a result of a histological switch. To the best of our knowledge, Park et al. published the first study in which a paired genomic analysis was performed [171]. To elucidate the underlying genomic changes that occur during transformation, they performed deep gene sequencing on pre and post samples from transformed SCC cases and identified genomic alterations in the *PI3K/AKT*/*mTOR* pathway [171]. These results are consistent with preclinical studies that support the hypothesis that the activation of this pathway is a core component of such a histological transformation. In particular, the loss of *LKB1* inhibits the negative regulation of mTOR and, consequently, leads to the activation of the pathway. An experimental *KRAS*-mutated *LKB1*-deficient model has been established in mice, showing that LKB1-deficient adenocarcinoma progressively transdifferentiates into SCC via an intermediate pathologically mixed adenosquamous histotype [172]. Interestingly, the authors demonstrated that LKB1 deficiency associated with lysil oxidase (LOX) reduction results in dramatic changes (extracellular matrix remodelling and p63 upregulation) and that adenocarcinoma cells might robustly and systematically transit to SCC [172,173]. The importance of the activated *PI3K/AKT/mTOR* pathway in the development of SCC tumorigenesis was also confirmed using an in vivo mouse model with biallelic *PTEN* and *LKB1* deficiency that developed pure SCC [174]. The activation of this pathway is an additional mechanism that could lead to TKI resistance in *EGFR*-mutated lung adenocarcinomas and merits in-depth investigation also considering its therapeutic implications [171].

### 2.3. Sarcomatous/Sarcomatoid Transformation

Sarcomatous transformation has rarely been described in lung adenocarcinoma treated with targeted therapy. The sarcomatous transformation of epithelial neoplasms represents the process whereby the neoplastic cell acquires certain mutations responsible for the epithelial-mesenchymal transition (EMT). The mutations responsible for the EMT include: transcriptional factors involved in regulatory pathways, such as zinc finger E-box binding homeobox 1 (*ZEB1*) (*δEF1*, *ZFHX1A*), *ZEB2* (*SIP1*, *ZFHX1B*), snail family transcriptional repressor 1 (*SNAI1*) (Snail), *SNAI2* (Slug), twist Family BHLH Transcription Factor 1 (*TWIST*), and *E12/E47*; alternative splicing; chromatin remodelling and epigenetic modifications; post-translational regulation; expression of non-coding RNAs; and alternative splicing [175].

EMT may underlie phenotypic changes in *EGFR*-mutated lung adenocarcinomas treated with TKIs, although this has mostly been demonstrated in in vitro or cell-line studies [81,176] and, rarely, in clinical settings, based mostly on case series or case reports [147,157,158,160,165]. The largest case series comprises six cases of lung adenocarcinomas (five with classic *EGFR* mutations and one with a *ROS1* rearrangement) that underwent a sarcomatoid transformation. All chemo-naïve cases shared the typical adenocarcinoma pathological findings (acinar, micropapillary, or solid/cribriform patterns, expression of E-cadherin and negativity for vimentin). Conversely, TKI-resistant tumours showed histologic features of sarcomatoid transformation, such as giant cell features, loose cellular cohesion, lesser expression of TTF1 and cytokeratin, and opposite staining patterns of e-Cadherin and Vimentin. The median interval from initial diagnosis to sarcomatoid transformation was 31.5 months with a median survival of 2.5 months. Moreover, the authors also detected high MET expression and *MET* copy number gain in five cases with *EGFR* mutations treated with EGFR TKIs [157]. These observations are consistent with the evidence of a high frequency of *MET* mutations (both exon 14 skipping and amplification) in lung sarcomatoid carcinomas, although the molecular mechanisms behind this link have not been elucidated. Determining the role of *MET* alterations would pave the way for the therapeutic application of MET inhibitors in resistant tumours with sarcomatous transformation. E-cadherin, a type of cell adhesion molecule fundamental in the formation of adherent junctions of epithelial cells, plays a central role in EMT. Some evidence suggests a link between E-cadherin expression and EGFR TKI sensitivity in NSCLC. Since the zinc finger transcriptional repressor, ZEB1, inhibits E-cadherin expression by recruiting histone deacetylases (HDAC), therapies based on HDAC inhibitors in association with TKIs have also been hypothesised [177]. However, more recent studies have observed that cadherin depletion does not represent a sufficient condition for the lack of sensitivity to TKIs in chemo-naive NSCLC, thus suggesting complex molecular mechanisms in which an imbalance of expression of epithelial markers in favour of mesenchymal ones (ZEB1, ZEB2, SNAI1, SNAI2, TWIST, and E12/E47) is responsible for EMT-driven resistance [178,179]. Another proposed mechanism is the negative influence of pro-EMT factors on apoptotic pathways following TKI treatment. Indeed, it has been observed that the inhibition of pro-apoptotic protein Bcl2-interacting mediator of cell death (BIM) mediated by ZEB1 [180] and TWIST1 [181] may represent a mechanism by which EMT pathways make NSCLCs resistant to TKIs. This could lead to the development of therapies based on Bcl-2 homology 3 (BH3) mimetics which may directly activate apoptosis by binding and inhibiting select antiapoptotic Bcl-2 family members [182]. Several studies have additionally reported that micro-RNAs may mediate the overexpression of pro-EMT factors in lung cancer by modulating the expression of pro-EMT factors [183,184,185].

Interpretation of the molecular mechanisms underlying the EMT may even be more complex considering the possible involvement of paracrine action of other cellular elements, for example cancer-associated fibroblasts (CAFs) that are part of the tumour microenvironment. A recent study found that CAFs could induce EMT in cell lines of *EGFR*-mutated NSCLC resistant to EGFR TKI gefitinib, particularly when there was an increment in the expression of Annexin A2, whose knockdown was found to completely reverse EMT phenotype and gefitinib resistance induced by CAFs [186].

## 3. Use of Liquid Biopsy in Monitoring Therapeutic Resistance

In the medical community, it is now well recognised that liquid biopsy (LB) is an attractive tool to use in daily practice for the detection of predictive biomarkers in advanced non-squamous non-small cell lung carcinoma (aNS-NSCLC) [187,188]. Currently, LB cannot replace a tissue biopsy for lung cancer diagnosis and for the detection of different molecular alterations for targeted immunotherapy [189,190,191]. Therefore, even if some investigators strongly believe in a “plasma-first” approach in aNS-NSCLC, the sensitivity for the detection of some genomic alteration (notably gene fusion and the gene amplification) is higher from tissue than from LB [151,192]. However, LB is very important for diagnostics when a tissue biopsy is unavailable [187,188].

Awareness of the importance of performing LB for NS-NSCLC patients at progression, notably in patients treated by a TKI, is progressively growing in routine clinical practice [187,188,193,194,195]. Indeed, LB is a relatively easy tool to use since it is non-invasive, repeatable, and cost-effective with no need for patient hospitalisation [196]. One of the most attractive features of LB is its usefulness for tracking the onset during tumour progression of a resistance mechanism in TKI-treated patients by genomic alteration assessment of circulating free DNA (cf-DNA) through blood sample monitoring. This usefulness is best demonstrated by an initial search for the appearance of the *EGFR* T790M mutation in cf-DNA in patients treated using first- or second-line generation TKIs [195,197]. The detection from LB of the *EGFR* T790M mutation, which is present in around 50% of patients progressing through the first or second generation of TKIs, led to the administration of a third generation of TKIs, essentially the osimertinib therapy [187,195]. Initially, this mutational detection was considered to have the advantage of allowing for a single targeted gene-sequencing test using a reverse transcriptase-polymerase chain reaction method [198,199,200]. The strong efficacy of osimertinib in first-line therapy was subsequently found to cause the appearance of other different resistance mechanisms [187,188,195,201,202].

In addition to detection of the onset of a secondary *EGFR* mutation (namely the C797S mutation, less frequent than the *EGFR* T790M mutation) as an indication of a new mechanism of osimertinib resistance, other genomic alterations occurring in different genes—notably in *MET*, *KRAS*, *BRAF*, or *RET*—can also be detected in cf-DNA in osimertinib-treated patients at tumour progression [195,202,203,204]. This highlights the necessity of using an NGS approach to simultaneously evaluate the status of several genes [205,206]. Similarly, patients treated with ALK inhibitors systematically develop resistance mechanisms, typically after several months of treatment [195,207,208]. Some of these latter mechanisms can be identified in cf-DNA, notably those occurring in different genes such as *ALK*, but also in *MET* and other genes [195,208]. *ALK* mutation is the main mechanism of resistance to ALK TKI treatments [195,207,208,209]. Today, many of these *ALK* mutations are identified, depending on the ALK TKI [195,207]. When identified, these *ALK* mutations may lead to the administration of a new ALK TKI [187,195,207]. More exceptionally, *ROS1*-rearranged tumours treated with crizotinib or entrectinib can have a resistance mechanism detected in cf-DNA, notably a *ROS1* mutation [210,211].

It is important to note that some genomic alterations occurring at progression in EGFR, ALK and ROS1 positive-treated patients can be more difficult to detect in cf-DNA than in tissue biopsies [203,207]. Indeed, gene amplification (such as *MET* amplification) and gene rearrangement (such as *EGFR*, *RET*, and *BRAF* rearrangements) sometimes cannot be detected in LB but can be detected in matched tissue biopsies [188,195,204]. This fact highlights that the sensitivity of genomic alteration detection in blood samples, in particular gene amplification and gene fusion, can be much lower than in tissue samples, and that a negative result in cf-DNA should lead to performing a systematic NGS in nucleic acids extracted from a tissue re-biopsy [188,212].

More recently, new targeted therapies received FDA and EMA approval for advanced NS-NSCLC first-line treatment [211]. These drugs target *NTRK* or *RET* rearrangement, but also *MET* amplification or *MET* exon 14 mutations [211]. Different genomic alterations on these latter genes or other genes may occur at tumour progression and could be potentially detected in LB as well. Additionally, promising molecules targeting *KRAS* mutations (in particular the *KRAS* G12C mutation), and *HER2* mutations may soon be available for therapeutic strategies in advanced NS-NSCLC [211]. Clinical trials using these new molecules showed that at tumour progression, patients developed different mechanisms of resistance, and some of them could be identified in cf-DNA [67,213].

Considering the increasing number of genomic alterations associated with a treatment resistance that must be identified at tumour progression, it is now obvious that the only molecular biology technology to be used in cf-DNA analysis is the NGS method [206]. In this context, different gene panel sizes are commercially available and the question becomes what size of gene panels (between 300 and 500 genes) should be used use in this context? [206]. Currently, these large gene panels can be used in external private platforms and very rarely in some in-house platforms equipped with sequencing devices to run such large panels [206]. The challenge is to achieve a turnaround time (TAT) for obtaining results according to international guidelines [214]. It is clear that well-controlled in-house workflow testing allows for a shorter TAT than those associated with an external procedure for NGS testing. Using a medium-sized panel (up to 50 genes), in-house platforms can be an effective way to determine the great majority of genomic alterations associated with a mechanism resistant to the targeted therapies that are currently available or in development.

It is noteworthy that some mechanisms resistant to targeted therapies, such as histological subtype transformation in SCLC or in SCC and the onset of an epithelial to mesenchymal transformation phenotype [195,203,207], cannot presently be detected in LB. Thus, in cases of tumour progression a tissue re-biopsy needs to be used to search for some of these mechanisms. Finally, in the near future, LB could be a promising approach to monitor some resistance mechanisms of immunotherapy at tumour progression, even if no biomarkers are currently used in daily practice for this purpose [200,215,216,217].

To sum up, most of the resistance mechanisms of NS-NSCLC occur at tumour progression under specific treatment, but some of them could be detected even at baseline, and in this context, LBs can also be useful tools for a better understanding of the complexity of lung cancer biology and for finding the best therapeutic strategy for NSCLC [196].

## 4. Conclusions

The incidence of drug resistance in switched oncogene addicted NSCLCs is progressively increasing. Although an increase in cases linked to a greater clinical surveillance cannot be excluded, emerging evidence of drug resistance certainly constitutes a critical barrier to molecular target treatment. Key unmet needs include a correct diagnostic approach to and management of patients. Researchers involved in this field need to intensify clinical and translational studies to better understand the relevant molecular and cellular mechanisms.
